# Sex and gender differences and biases in artificial intelligence for biomedicine and healthcare

**DOI:** 10.1038/s41746-020-0288-5

**Published:** 2020-06-01

**Authors:** Davide Cirillo, Silvina Catuara-Solarz, Czuee Morey, Emre Guney, Laia Subirats, Simona Mellino, Annalisa Gigante, Alfonso Valencia, María José Rementeria, Antonella Santuccione Chadha, Nikolaos Mavridis

**Affiliations:** 1 Barcelona Supercomputing Center (BSC), C/ Jordi Girona, 29, 08034 Barcelona, Spain; 2 Telefonica Innovation Alpha Health, Torre Telefonica, Plaça d’Ernest Lluch i Martin, 5, 08019 Barcelona, Spain; 3 The Women’s Brain Project (WBP), Guntershausen, Switzerland; 4 Wega Informatik AG, Aeschengraben 20, CH-4051 Basel, Switzerland; 5 Research Programme on Biomedical Informatics (GRIB), Hospital del Mar Research Institute and Pompeu Fabra University, Dr. Aiguader, 88, 08003 Barcelona, Spain; 6 Eurecat - Centre Tecnològic de Catalunya, C/ Bilbao, 72, Edifici A, 08005 Barcelona, Spain; 7 eHealth Center, Universitat Oberta de Catalunya, Rambla del Poblenou, 156, 08018 Barcelona, Spain; 8 ICREA, Pg. Lluís Companys 23, 08010 Barcelona, Spain; 9 Interactive Robots and Media Laboratory (IRML), Abu Dhabi, United Arab Emirates

**Keywords:** Biomarkers, Computational models, Risk factors, Medical ethics

## Abstract

Precision Medicine implies a deep understanding of inter-individual differences in health and disease that are due to genetic and environmental factors. To acquire such understanding there is a need for the implementation of different types of technologies based on artificial intelligence (AI) that enable the identification of biomedically relevant patterns, facilitating progress towards individually tailored preventative and therapeutic interventions. Despite the significant scientific advances achieved so far, most of the currently used biomedical AI technologies do not account for bias detection. Furthermore, the design of the majority of algorithms ignore the sex and gender dimension and its contribution to health and disease differences among individuals. Failure in accounting for these differences will generate sub-optimal results and produce mistakes as well as discriminatory outcomes. In this review we examine the current sex and gender gaps in a subset of biomedical technologies used in relation to Precision Medicine. In addition, we provide recommendations to optimize their utilization to improve the global health and disease landscape and decrease inequalities.

## Introduction

Precision Medicine, as opposed to the preponderant one-size-fits-all approach, attempts to find personalized preventative and therapeutic strategies by taking into account differences in genes, environment and lifestyle, throughout the lifespan. The value and impact of this approach makes Precision Medicine one of the most promising health initiatives in our society^1^.

Both biological (sex) and socio-cultural (gender) aspects (see Supplementary Note 1 “Sex and gender”) constitute relevant sources of variation in a number of clinical and subclinical conditions, affecting risk factors, prevalence, age of onset, symptomatology manifestation, prognosis, biomarkers and treatment effectiveness^2^. Evidence of sex and gender differences has been reported in chronic diseases such as diabetes, cardiovascular disorders, neurological diseases^3^, mental health disorders^4^, cancer^5^, autoimmunity^6^, as well as physiological processes such as brain aging^7^ and sensitivity to pain^8^. Moreover, differences in lifestyle factors that are associated with sex and gender, such as diet, physical activity, tobacco use and alcohol consumption, also correlate with the epidemiology of diseases^9–11^. Nonetheless, there are still open questions regarding health differences across the gender spectrum, reflected by the scarcity of studies dedicated to intersex, transgender and nonbinary individuals^12,13^. Initiatives, such as the Global Trans Research Evidence Map^14^, foster research access in this area to improve our understanding of the effects of medical interventions on health and life quality across the gender spectrum. Additionally, such clinical differences are accompanied by sex and gender gaps in the use and access of medical services and tools as well as affordability to medical costs^15^.

**Fig. 1:**
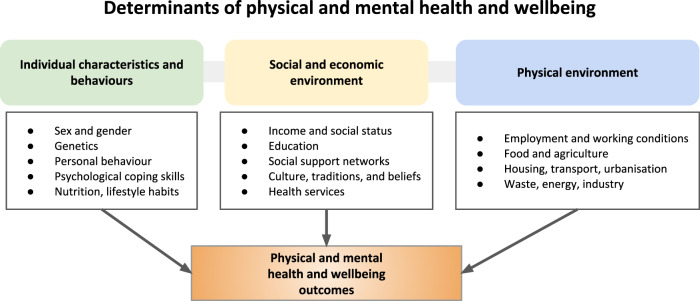
The key determinants of health. Health and wellbeing of individuals and communities are influenced by several factors, which include the person’s individual characteristics and behaviours and the socio-economic, and physical environment, according to the World Health Organization (WHO) (www.who.int/hia/evidence/doh/en/). Sex and gender differences interact with the whole spectrum of health determinants.

The study of sex and gender differences represents an increasingly significant line of research^16^, involving all levels of biomedical and health sciences, from basic research to population studies^17^, and also fueling debate regarding its sociological implications^18,19^. Observed sex and gender differences in health and wellbeing are influenced by complex links between both biological and social-economic factors (see Fig. 1), which are often surrounded by confounding variables such as stigma, stereotypes, and the misrepresentation of data. Consequently, health research and practices can be entangled with sex and gender inequalities and biases^20^.

In recent years, the social awareness of such biases has increased and they have become even more evident with the introduction of widespread advance in biomedical artificial intelligence (AI). In this regard, one could argue that AI technologies act as a double-edged sword. On one hand, algorithms can magnify and perpetuate existing sex and gender inequalities if they are developed without removing biases and confounding factors. On the other hand, they have the potential to mitigate inequalities by effectively integrating sex and gender differences in healthcare if designed properly. The development of precise AI systems in healthcare will enable the differentiation of vulnerabilities for disease and response to treatments among individuals, while avoiding discriminatory biases.

The purpose of this review is to highlight the main available biomedical data types and the role of several AI technologies to understand sex and gender differences in health and disease. We address their existing and potential biases and their contribution to create personalized therapeutic interventions. We examine the sex and gender issues involved with the generation and collection of experimental, clinical and digital data. Furthermore, we review a number of technologies to analyze and deploy this data, namely Big Data Analytics, Natural Language Processing and Robotics. Those technologies are becoming increasingly relevant for Precision Medicine while being exposed to potential sex and gender biases. In addition, we surveyed Explainable AI and algorithmic Fairness, which ensure the trustworthy delivery of AI solutions that can account for sex and gender differences in the patient’s wellbeing. Finally, we provide a summary to incorporate the sex and gender dimension into biomedical research and AI technologies to accelerate the developments that will enable the creation of effective strategies to augment populations’ health and wellbeing.

### Desirable vs. undesirable biases

**Fig. 2:**
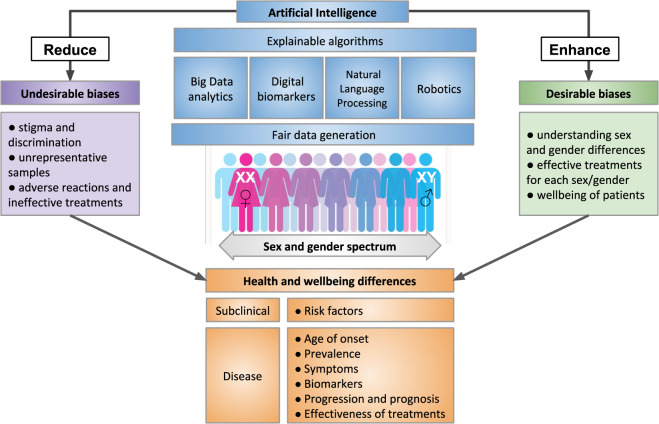
Desirable and undesirable biases in artificial intelligence for health. Fair data generation and explainable algorithms are fundamental requirements for the design and application of artificial intelligence to optimize for health and wellbeing across the sex and gender spectrum. This will facilitate the reduction of undesirable biases that propagate inequity and discrimination, and will promote desirable differentiations that help develop Precision Medicine.

Despite the fact that the term “bias” has gained a negative connotation due to its association to unfair prejudice, the differential consideration and treatment to specific biomedical aspects is a necessary course of action in the context of Precision Medicine. Therefore, here we defined two main categories of sex and gender biases: desirable and undesirable (see Fig. 2). The difference between them is found in the impact that these biases have on the patients’ wellbeing and healthcare access.

A desirable bias implies taking into account sex and gender differences to make a precise diagnosis and recommend a tailored and more effective treatment for each individual. This represents a much more accurate approach than collapsing all sex and gender categories into a single one, such as data generated from mixed sex or gender cohorts^16^. Table 1 reports illustrative examples of clinical conditions and biomedical techniques in which desirable biases would be beneficial for both basic and clinical research as well as diagnosis and treatment.

**Table 1:** Illustrative examples of clinical conditions and studies in which desirable biases would be beneficial for both basic and clinical research as well as diagnosis and treatment.

Clinical conditions and studies	Current status without the desirable bias	Utility of the desirable bias
Autistic spectrum disorder	There is a current lack of consideration of the demonstrated age-dependent sex differences in the symptomatology related with impairments in social communication and interaction, expressive behaviour, reciprocal conversation, non-verbal gestures for diagnostic purposes^123^.	Differential diagnostic criteria for males and females could facilitate the identification of the clinical diagnosis leading to appropriate treatment.
Cardiovascular disorders	Although it has been documented that men and women respond differently to many cardiovascular medications such as statins, angiotensin-converting enzyme inhibitors and β-Blockers among others, adopted treatments do not consider sex differences^124^.	Making prescriptions according to the sex of the patient could lead to improved health benefits.
	Despite the fact that Coronary heart disease (CHD) is the leading cause of death among women^125^, the majority (67%) of patients enroled in clinical trials for cardiovascular devices are male^126^.	The application of a desirable bias towards women would lead to a more accurate representation of sex differences in clinical research.
Genome-wide association studies (GWAS)	Most of genome-wide association studies (GWAS) focus on white male subjects^127^ and those that explore sex differences in complex traits are scarce^128^.	The introduction of desirable biases to deliberately include female subjects and other ethnicities in GWAS could lead to better account for potential sex differences in disease that are currently unknown because of being overlooked.
Human immunodeficiency virus (HIV)	The observed lower female representation in HIV clinical trials depends, among other factors, from the disadvantaged awareness about treatment and enrolment options compared with men^129–131^.	Promoting empowerment initiatives in those patients with disadvantages will increase their exposure to treatment options and clinical trial enrolment.

Conversely, an undesirable bias is that which exhibits unintended or unnecessary sex and gender discrimination. This occurs when claims are made in relation with sex or gender and medical conditions despite the lack of exhaustive evidence to support them or based on skewed evidence.

For instance epidemiological studies indicate that there is a higher prevalence of depression among women, however, this may result from a skewed diagnosis due to clinical scales of depression measuring symptoms that occur more frequently among women^21^. Another source of undesirable bias is the misrepresentation of the target population, leaving minorities out. An example of this is the case of the insufficient representation of pregnant women in psychiatric research^22^.

There are multiple sources of undesirable biases that could accidentally be introduced in AI algorithms^23^ (see Table 2). The most common one is the lack of a representative sample of the population in the training dataset. In some cases, a bias may exist in the overall population as a consequence of underlying social, historical or institutional reasons. In other cases, an algorithm itself, and not the training dataset, can introduce bias by obscuring an inherent discrimination or inducing an unreasoned or irrelevant selectivity.

**Table 2:** Source of undesirable bias in Artificial Intelligence with examples in health research and practice.

Source of bias in artificial intelligence	Description
Historical bias	Arises even if the data is perfectly measured and sampled, when the world as it is leads a model to produce outcomes that are not desired. e.g. incorrectly assuming that HIV is inherently linked to homosexual and bisexual men as its prevalence is higher in this population^132^.
Representation bias	Occurs when certain parts of the input space are underrepresented. e.g. European male populations are the primary focus in genomics research and its derived clinical findings, neglecting other ethnicities and populations^133^.
Measurement bias	Occurs when measured data are often proxies for some ideal features and labels. e.g. the use of clinical, social, and cognitive variables to detect the prodromal phase in schizophrenia and other psychotic disorders despite of observed sex differences in the expression of those symptoms and their associated risk for psychosis^134^.
Aggregation bias	Arises when a one-size-fits-all model is used for groups with different conditional distributions. e.g., for the diagnosis and monitoring of diabetes, haemoglobin A1c (HbA1c) levels are routinely used, despite of differences associated with ethnicities^135^ and gender^136^.
Evaluation bias	Occurs when the evaluation and/or benchmark data for an algorithm does not represent the target population. e.g. underperformance of commercial facial recognition algorithm in dark-skinned female faces as most benchmark face image datasets come from white men^137^.
Algorithmic Bias	Occurs when bias is introduced in the algorithm consciously or unconsciously in ad-hoc solutions. e.g. by using health care cost as a proxy feature for health status without correcting for existing inequalities in health access, a commercial algorithm to predict health care needs was found to exhibit significant racial discrimination^138^.

## Sources and types of health data

### Experimental and clinical data

In the early days of biomedical research and drug discovery, sex-specific biological differences were neglected and both experimental and clinical studies were fundamentally focused on male experimental models or male subjects^24^. Even nowadays, male mouse models are overall more represented than female models in basic, preclinical, and surgical biomedical research^25^. A recent analysis of data on 234 phenotypic traits from almost 55,000 mice showed that existing findings were influenced by sex^26^. The lack of representation of female models and patients is partly due to technical and bioethical considerations, such as the attempt to reduce the impact of estrous cycle in experimental studies and protective policies for women of childbearing age in clinical research. Consequently, some of the treatments that currently exist for several diseases are not adequately evaluated in women^27,28^ who are likely to be underrepresented in clinical trials^29,30^, especially in Phases I and II^31,32^.

Differences in the physiology of sexes^33^ might translate into clinically relevant differences in pharmacokinetics and pharmacodynamics of drugs. These differences, taken together with the underrepresentation of women in clinical trials, can explain why women typically report more adverse event reactions compared with men^34^. An illustrative example of the discrepancy between sexes in clinical trials is zolpidem, a sleep medication^35^, which shows slower drug metabolization and high secondary effects in women, increasing their health risks compared with men^34,36,37^. In 2013, the FDA recommended a weight-based dosing zolpidem for women due to potential sex-specific impairments^38^, proving how a stratified consideration of sexes enables a better understanding of differential drug toxicity. The design of preclinical and clinical studies should have a sex and gender-based approach in order to reduce the time to translate research into clinical practice, as well as to understand and implement precise pharmacological guidelines^39^.

Accounting for sex and gender differences leads to a better understanding of the pharmacodynamic and pharmacokinetic action of a drug. It also carries substantial economic implications^40^ as conducting studies on large population-based trials is generally more expensive^41^, and often requires post-trial analyses to identify and categorise the factors that explain the varying drug response across individuals.

In summary, although there is a significant gap between two sexes on the availability of clinical data and the knowledge on the effects of drugs, recent clinical guidelines and initiatives hint to a fairer landscape that accounts for sex differences in biomedical research and clinical practice.

### Digital biomarkers

Digital biomarkers are physiological, psychological and behavioral indicators based on data including human-computer interaction (e.g. swipes, taps, and typing), physical activity (e.g. gait, dexterity) and voice variations, collected by portable, wearable, implantable or even ingestible devices^42^. They can facilitate the diagnosis of a condition, the assessment of the effects of a treatment and the predicted prognosis for a particular patient. In addition, some digital biomarkers can inform on patient adherence to treatment.

There are many digital biomarkers that are currently being developed or already approved or cleared by the U.S. Food and Drug Administration (FDA) for use cases such as risk detection, diagnosis, and monitoring of symptoms and endpoints in clinical trials^42^ (see Table 3).

**Table 3:** Categories of digital biomarkers.

Category	Definition	Corresponding digital biomarker examples
Susceptibility and risk biomarker	A biomarker that indicates the potential for developing a disease or medical condition in an individual who does not currently have clinically apparent disease or medical condition.	^a^Detect cognitive changes in healthy subjects at risk of developing Alzheimer’s disease using a video game platform^139^.
Diagnostic biomarker	A biomarker used to detect or confirm the presence of a disease or condition of interest or to identify individuals with a subtype of the disease.	^a^Diagnose ADHD in children using eye vergence metrics^140^. ^a^Detect depression and Parkinson’s disease using vocal biomarkers^141^. ^a^Diagnose asthma and respiratory infections using smartphone-recorded cough sounds^142^.
Monitoring biomarker	A biomarker measured serially for assessing the status of a disease or medical condition or for evidence of exposure to (or effect of) a medical product or an environmental agent.	^a^Quantify Parkinson’s disease severity using smartphones and machine learning^143^. ^b^Track time and location of short-acting beta-agonist inhaler use through an attached wireless sensor^144^. ^a^Detection of nocturnal scratching movements in patients with atopic dermatitis using accelerometers and recurrent neural networks^145^. ^b^Measurements of sympathetic nervous impulses at the skin and inference of parasympathetic activity from heart rate variation to detect tonic-clonic epileptic seizures and immediately alert care providers^146^. ^b^Portable electrocardiogram sensor associated to a smartphone app to monitor atrial fibrillation, bradycardia, tachycardia or normal heart rhythm and inform the clinician^147^. ^a^Measure adherence in treatment of schizophrenia and bipolar disorder with an ingestible digital pill^148^.
Endpoint digital biomarkers in clinical trials	Endpoints generated by the use of mobile technologies in clinical setting.	^a^Accelerometer-derived motor abnormalities for use in Parkinson’s disease^47^. ^b^Monitoring of multiple sclerosis patients with digital technologies by using active and passive tests (ClinicalTrials.gov Identifiers: NCT03523858; NCT02952911) ^b^Virtual Reality Functional Capacity Assessment Tool as co-primary and secondary endpoint in schizophrenia and major depressive disorder^139^.

^a^Digital biomarker under development (in feasibility/exploratory stages).

^b^Digital biomarker in use in a clinical trial or an FDA cleared/approved digital health product, or a digital health app in use not requiring approval.

A particular therapeutic area where digital biomarkers are becoming beneficial is that of neurological and mental health disorders. Since digital devices can acquire health related data in real-time, they can enable a continuous monitoring of an individual’s health parameters in a cost-effective way that is more granular, ecological and objective than the currently clinically used self-reports, questionnaires or psychometric tests. Digital biomarkers are becoming especially relevant for those clinical conditions where small fluctuations in daily symptoms or performance are clinically meaningful. This is the case, for example, of early detection of neurodegenerative disorders such as Alzheimer’s disease (AD), in which key indices of preclinical stages are cognitive, motor and sensory changes that occur 10 or 15 years prior to its effective diagnosis^43,44^.

Despite the progress that has been made on digital biomarkers in the last years, sex and gender differences in these indices of health and disease have not been examined yet. Considering that several studies have shown that there are significant sex differences on neurodegenerative, physiological and cognitive aspects during the preclinical stages of AD^45^, it is reasonable to expect that further sex differences will be found in the digital biomarkers for this and other clinical conditions.

In some cases the analysis of sex differences on digital biomarkers is prevented by undesired biases in the datasets used by the models that provide the health indicators. For instance, current studies that test digital biomarkers are often performed with small sample sizes in the range of tens to hundreds of subjects and tend to show insufficient demographic information on sex and gender^46^. For example, in a study assessing digital biomarkers for Parkinson’s disease (PD), only 18.6% were women^47^. As a consequence, if an algorithm is trained with a dataset overrepresented by male patients, it may lead to a more accurate detection of those symptoms that are more frequently manifested by male PD patients (rigidity and rapid-eye movement) in comparison to those symptoms that are more frequently manifested by female PD patients (dyskinesias and depression)^48^.

In other cases, the undesired biases arise from the digital device itself, such as in the case of a pulse oximetry which showed errors in the predicted arterial oxyhemoglobin saturation associated with sex and skin colour of the subjects^49^.

**Fig. 3:**
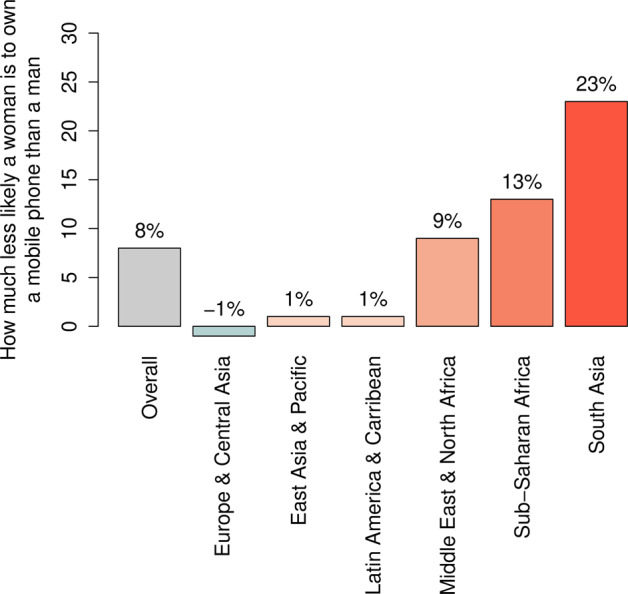
The digital divide in access to mobile technology around the globe. The bar plot reports how less likely a woman is to own a mobile phone than a man, according to a survey analysis on mobile ownership conducted by the Global System for Mobile Communications Association (GSMA) in low- and middle-income countries (LMIC) in 2019, by geographical area (source: GSMA “The Mobile Gender Gap Report 2020”^51^). For instance, in South Asia women are 23% less likely than men to be the owner of a mobile phone, while in Europe and Central Asia women are 1% more likely to be the owner of a mobile phone. Across LMICs (“Overall”), women are 8% less likely than men to own a mobile phone.

An additional source of undesired biases in digital biomarkers is the unbalanced access, and use of digital devices among people with different sexes and genders as well as education and income levels and age^50^. In fact, in low and middle income countries, women are 10% less likely to own smartphones (see Fig. 3) and 26% less likely to use the internet compared with men, and 1.2 billion women do not even have access to mobile internet^51^. This creates uneven datasets that promote misrepresentation of digital biomarkers. Awareness and efforts into the identification of sex and gender differences in digital biomarkers will lead to more accurate indicators for prevention and diagnosis of disease, as well as more effective treatment monitoring.

## Technologies for the analysis and deployment of health data

### Big Data analytics

Big Data analytics is a body of techniques and tools to collect, organize and examine large amounts of data. Common Big Data analytics processes and approaches include the creation of data management infrastructures and the application of data-driven algorithms and AI solutions^52^. Biomedical and clinical Big Data have the potential of providing deeper insights into health and disease at an unprecedented scale. Moreover, the availability of longitudinal health Big Data enables the characterization of the transitions between health and disease states as well as their similarities and differences among sexes and genders. Large international research infrastructures, such as ELIXIR^53^ and NIH Big Data to Knowledge (BD2K)^54^, provide robust, long-term sustainable biomedical resources that will enable identifying differential patterns for health and disease transitions including the sex and gender dimension.

For instance, data from GWAS targeting smoking behaviour have shown sex-associated genetic differences that influence smoking initiation and maintenance^55^. Interestingly, these differences complement the differential effectiveness of tobacco control initiatives based on the sex of the individuals that receive the preventative messages^56^. Similarly, genomic studies in large human cohorts revealed chromosomal factors related to sex differences in excess body fat accumulation^57^, interlinking recent insights on obesity from different Big Data types such as social media, retail sales, commercial data, geolocalization, transport and digital devices^58^.

Big Data analytics focused on health under the sex and gender lens are carried out worldwide by several initiatives such as Data2x (www.data2x.org). This collaborative platform explores female wellbeing through statistical analysis of data covering demography, education, health, geolocation, in order to map indices disaggregated by gender.

For instance, significant sex differences in behavioral and social patterns related with communication such as the number and duration of phone calls and the degree of social networking callers have been observed^59^. Furthermore, quantitative analysis into sex and cultural differences uncovered associations with mental health and social networks^60^, showing men express higher negativity and lower desire of social support on social media compared with women.

Awareness of sex and gender differences through biomedical Big Data could lead to a better risk stratification. For example, a query of sex and gender differences in heart diseases revealed that in women enhanced parasympathetic and decreased sympathetic tones appear to be greater and also defensive during cardiac stress^61^, while key reproductive factors associated with coronary heart disease only modestly improve risk prediction^62^.

The caveat of these resources is that the exploitation of their biomedical Big Data can magnify existing undesirable biases, for instance by introducing inferential errors due to sampling, measurement, multiple comparison, aggregation, and systematic exclusion of information^63^. For example, biases may be introduced in clinical decision support algorithms that rely on data obtained from the large reservoirs of electronic health records (EHRs), which may display missing data, unbalanced representation, and implicit selectivity in patient factors such as sex and gender^24^.

In agreement with the Findability, Accessibility, Interoperability, and Reusability (FAIR) recommendations for responsible research and gender equality^64^, biomedical Big Data requires innovative procedures for bias corrections^65^, including sex and gender bias, as well as algorithm interpretability^66^ (see Valuable outputs of health data), facing mounting pressure in data processing and privacy with the pursuit of “equal opportunity by design”^67^. Fair big data analytics will facilitate the identification of sex and gender differences in health as well as accurate indicators for prevention and diagnosis, and effective treatment.

### Natural Language Processing

Natural Language Processing (NLP) consists of computational systems aimed at understanding and manipulating written and spoken human language for purposes like machine translation, speech recognition and conversational interfaces^68^.

In relation to biomedical research, NLP techniques allow processing of voice recordings and transcripts as well as large volumes of scientific knowledge accumulated in the textual forms, such as biomedical literature, electronic medical records, clinical trials and pathology reports. This automatic processing enables, for instance, the creation of major knowledge bases such as NDEx (https://home.ndexbio.org/), OncoKB (https://oncokb.org), and Literome^69^.

As for Precision Medicine, these technologies allow to make predictions that can contribute to clinical decisions, such as diagnosis, prognosis, risk of relapse, and symptomatology fluctuations in response to treatments. Examples of applications of NLP to Precision Medicine comprise the identification of personalised drug combinations^70^, the knowledge-based curation of clinical significance of variants^71^, and patient trajectory modelling from clinical notes^72^. Activities to overcome some of the main challenges in NLP, such as complex semantics extraction and reasoning, entail automated curation efforts, such as Microsoft Project Hanover (https://www.microsoft.com/en-us/research/project/project-hanover/), and evaluation campaigns, such as BioCreative^73^.

The sex and gender dimension is crucial for the development of effective NLP solutions for health since multiple sex and gender differences have been documented in written and spoken language^74^. In fact, major differences are observed in dialogue structure^75^, word reading^76^, and even in children’s linguistic tasks^77^. Although the reasons for the differential use of language between men and women needs further investigations^78^, the existence of such differences can either facilitate or complicate the development of NLP technologies. For instance, while it is possible to accurately categorize texts based on the author’s gender^79^, performances of sentiment analysis of male- and female-authored texts are extremely variable^80^ and potentially biased^81^. Thus, knowing the sex and gender of the author enables a better targeted prediction of symptoms conveyed through natural language (text or speech). An example of this is the case of personalised healthcare for transgender and gender nonconforming patients based on EHRs analysis^82^.

In the context of NLP for voice recognition, the relevance of sex differences is evident in applications such as the prediction of suicidal behaviour^83^, especially considering the reported inconsistent and incomplete responses by popular conversational agents (Apple, Samsung, Microsoft) to suicidal ideation^84^.

A case of undesirable biases in NLP is the use of text corpora containing imprints of documented human stereotypes that can propagate into AI systems^85^. For instance, dense vector representations called word embeddings^86^ are able to capture semantic relationships between words, such as sex, gender and ethnic relationships^87^, thus absorbing biases existing in the training corpus^88^. Methods for bias mitigation in NLP have been recently reviewed, including learning gender-neutral embeddings and tagging the data points to preserve the gender of the source^89^.

A flourishing area of NLP is that of medical chatbots, aiming to improve users’ wellbeing through real-time symptom assessment and recommendation interfaces. A dialogue of a chatbot can be modelled with available metadata to adjust to features of the replier in terms of gender, age, and mood^90^. In the context of mental health, medical chatbots include Woebot, which proved to relieve feelings of anxiety and depression^91^, and Moodkit, which recommends chatting and journaling activities through text and voice notes^92^. Although both proved to be effective in clinical trials, the lack of data on their long-term effects is raising certain concerns. These include the risk of oversimplifying mental conditions and therapeutic approaches, without considering potentially important factors such as sex and gender differences in non-verbal communication.

Of note, affective computing (i.e. passively estimating human emotional states in real-time) has started to be integrated in automated systems for educational and marketing purposes^93^, as well as voice-activated assistants for mental health support like Mindscape (www.cultmindscape.com). In this regard, potential undesirable biases may undermine the automatic detection of sex-associated speech fluctuation in cognitive impairment^94^.

In the development and application of biomedical NLP systems, awareness of sex and gender differences is a crucial step in our understanding of women’s and men’s relative use of language, which could lead to a better patient management and more effective risk stratification.

### Robotics

Robots can serve a diverse range of roles in improving a human’s tasks, health and quality of life. In the context of Precision Medicine robots are expected to provide personalised assistance to patients according to their specific needs and preferences, at the right time and in the right way. Robotics for health are becoming increasingly impactful, in particular in neurology^95^, rehabilitation^96^, and assistive approaches for improving the quality of life of patients and caregivers^97^.

In a personalised robot-patient interaction both the gender of the patient and the “gender” of the robot have to be taken into account. While there is not a lot of research on how to personalise the behaviour of a robot (e.g. speech style) to an individual’s gender, several studies explored how the gendered appearance of a robot differentially affects human-robot interactions. For instance, a recent study revealed sex differences in how children interact with robots^98^ with implications for their use in paediatric hospitalization^99^.

The application of robots in human society makes the discussion on humanoids’ gender extremely relevant and significantly variable across cultures^100,101^. While some robots are genderless, such as Pepper (Softbank), ASIMO (Honda), and Ripley (MIT), others are designed to display explicit gendered features, such as the females Sophia (Hanson Robotics), Sarah the FaceBot^102^, and male Ibn Sina Robot, a culture-specific historical humanoid^103^. This opened a strong debate regarding the commonalities among humans and robots on physical, sociological and psychological gender^104^.

It has been demonstrated that the outcome of a humanoid robot’s task can be affected by its gender, as in the case of female charity robots receiving more donations from men in comparison to women^100^. Indeed, the fact that the traits of a gendered robot are developed in accordance with the perceived gender role of both the developer and the final user, could emphasize social constructs and stereotypes. Gender representation in robots should evade social stereotypes and serve functionally human-robot interactions^102^. An illustrative effort towards gender neutrality in robotics is the creation of a genderless digital voice (https://www.genderlessvoice.com/), designed using a gender-neutral frequency range (145–175 Hz).

Awareness of sex and gender differences in patients and in robots could lead to a better healthcare assistance and effective human-machine interactions for biomedical applications as well as a better translation of ethical decision-making into machines^105^.

## Valuable outputs of health technologies

### Towards explainable artificial intelligence

In the context of Precision Medicine, the expected outputs of AI models consist of predictions of risk and diagnosis of medical conditions or recommendations of treatments, with profound influence in people’s lives and health.

Despite the progress of AI models in recent years, the complexity of their internal structures has led to a major technological issue termed the ‘Black box’ problem. It refers to the lack of explicit declarative knowledge representations in machine learning models^106^, meaning their inability to provide a layman-understandable explanation and/or interpretation to respond to “how” or “why” questions regarding their output.

Getting an explicable justification of how and why these AI models reach their conclusions is now becoming more and more crucial since there is an increasing need to understand the specific parameters used to draw clinical conclusions with relevant impact on patients’ lives. Indeed, the EU directive 2016/680 General Data Protection Regulation (GDPR) states the “right to an explanation” about the output of an algorithm^107^.

In regards to the scope of this review, explainability in AI would help justify algorithms’ clinical predictions and recommendations when they are differential for patients with different sex and genders. On one hand, an explanation of the decisional process would enable to find potential mistaken conclusions derived by training an algorithm with misrepresented data. This will facilitate the identification of undesirable biases generally found in clinical data with unbalanced sex and gender representation. On the other hand, an explanation of the decisional processes will help the discovery of sex and gender differences in clinical data that is representative, therefore promoting the desired biases for personalised preventative and therapeutic interventions.

Different features such as interpretability and completeness (see Supplementary Note 2 “Explainable Artificial Intelligence”) in AI have been established as explainability requirements to contribute to relevant aspects of general medicine such as confidence, safety, security, privacy, ethics, fairness and trust.

The term explainable artificial intelligence (XAI) is used to refer to algorithms that are able to meet those requirements. XAI is a relatively young field of research and their applications so far have not been particularly involved with sex and gender differences.

An example of XAI is a recent study where a machine learning algorithm made referral recommendations on dozens of retinal diseases, highlighting the specific structures in optical tomography scans that could lead to ambiguous interpretation^108^. Another example is a deep learning model for predicting cardiovascular risk factors based on images of the retina, indicating which anatomical features, such as the optic disc or blood vessels, were used to generate the predictions^109^. XAI is also useful in basic research, for instance, efforts in creating “visible” deep neural networks that provide automatic explanations of the impact of a genotypic change on cellular phenotypic states^110^.

XAI represents a promising technology to assist in the identification of sex and gender differences in health and disease, and to dissociate the underlying sources from biased datasets or social inequalities.

### Bias detection frameworks for fairness

One of the main challenges to develop trustworthy AI is to define the meaning of fairness in the practice of machine learning^111^. Indeed, many approaches have been proposed to achieve fair algorithmic decision-making, some of which not always meet the expected outcome.

For instance, a widely used approach to ensure fairness in data processing is to remove some sensitive information, such as sex or gender, and all other possible correlated features^112^. However, if inherent differences exist in the underlying population, such as sex differences in disease prevalence, this procedure is undesirable as the outcome would be less fair towards specific minorities. Indeed, the learned patterns that apply to the majority group might be invalid for a minority one.

On the contrary, the explicit use of sex and gender information enables to reach an outcome that is fairer towards minorities, which is a desirable procedure when inherent differences exist. A theoretical implementation of such approach, also called fair affirmative action, has been proposed as an optimisation problem to obtain, at the same time, both group fairness (a.k.a statistical parity) and individual fairness^113^.

Although affirmative action represents a remedy for unfair algorithmic discrimination, ensuring the quality of the data used for algorithm training is also crucial. For instance, a study found that only 17% of cardiologists correctly identified women as having greater risk for heart disease than men^114^. Indeed, physicians are typically trained to recognise patterns of angina and myocardial infarction that occur more frequently in men, resulting in women being typically under-diagnosed for coronary artery disease^115^. Consequently, training an algorithm on available data on diagnosed cases could be influenced by an implicit sex and gender bias.

Fairness is highly context-specific and requires an understanding of the classification task and possible minorities. Awareness and deep knowledge of sex and gender differences as well as the related socio-economical aspects and possible confounding factors are of paramount importance to establish fairness in algorithmic development.

The development and application of fair approaches will be critical for the implementation of unbiased and interpretable models for Precision Medicine^106,116^. In this regard, the use of visualizations, logical statements, and dimensionality reduction techniques can be implemented in computational tools to achieve interpretability^23^.

Mitigating undesirable bias to achieve fairness might require an explicit instruction to the artificial learning engine including rules of appropriate conduct, as proposed in the domain of cognitive robotics^117^. In addition, caution should be used particularly with the unsupervised learning components of AI given the wide availability of biased data sets and self-learning algorithms. Recent developments in bias detection and mitigation also include methods such as adopting re-sampling^118^, adversarial learning^119^, and open-source toolkits such as IBM AI Fairness 360 (AIF360) (aif360.mybluemix.net) and Aequitas (dsapp.uchicago.edu/projects/aequitas).

## Discussion

Technological advances in machine learning and AI are transforming our health systems, societies, and daily lives^120^. In the context of biomedicine, such systems can sometimes either neglect desired differentiations, such as sex and gender, or amplify undesired ones, such as reinforcing existing socio-cultural discriminations that promote inequalities.

The ambitious goals set by Precision Medicine will be achieved using the latest advances in AI to properly identify the role of inter-individual differences. This will include the impact of sex and gender in health and disease, as well as eradicating existing undesirable sex and gender biases from data sets, algorithms and experimental design. The proper use of innovative technologies will pave the way towards tailored and personalised disease prevention and treatment, accounting for sex and gender differences and extending towards generalized wellbeing. Actions that foster the effective utilization of AI systems will not only enable the acceleration towards Precision Medicine, but most importantly, will significantly contribute to the improvement of the quality of life of patients of all sexes and genders.

Ethical standards will have to continue to be considered by governments and regulatory organisations to guarantee the preservation of personal data privacy and security as well as to determine the way new technological tools should be employed, data should be collected, and models improved^121,122^. Governments and regulatory organisations are establishing the guidelines for actions in this direction, such as the case of AI-WATCH (https://ec.europa.eu/knowledge4policy/ai-watch), an initiative of the European Commission to monitor the socio-economic, legal and ethical impact of AI and robotics.

Based on the information surveyed in this work, we provide the following recommendations to ensure that sex and gender differences in health and disease are accounted for in AI implementations that inform Precision Medicine:

1. Distinguish between desirable and undesirable biases and guarantee the representation of desirable biases in AI development (see Introduction: Desirable vs. Undesirable biases).
2. Increase awareness of unintended biases in the scientific community, technology industry, among policy makers, and the general public (see Sources and types of Health data and Technologies for the analysis and deployment of Health data).
3. Implement explainable algorithms, which not only provide understandable explanations for the layperson, but which could also be equipped with integrated bias detection systems and mitigation strategies, and validated with appropriate benchmarking (see Valuable outputs of Health technologies).
4. Incorporate key ethical considerations during every stage of technological development, ensuring that the systems maximize wellbeing and health of the population (see Discussion).

## Supplementary information

Supplementary Information

## Data Availability

No datasets were generated or analyzed during the current study.
