# Supplementary material for: Sex and gender differences and biases in artificial intelligence for biomedicine and healthcare
Source: NPJ Digit Med. 2020 Jun 1;3:81. doi: 10.1038/s41746-020-0288-5 (PMC7264169; doi:10.1038/s41746-020-0288-5)
Supplement: Supplementary file 1 — Supplementary Information [file 41746_2020_288_MOESM1_ESM.pdf]

Sex and gender differences and biases in Artificial Intelligence for Biomedicine and Healthcare

Davide Cirillo[1,*,#], Silvina Catuara-Solarz[2,3,#], Czuee Morey[3,4], Emre Guney[5], Laia Subirats[6,7], Simona Mellino[3], Annalisa Gigante[3], Alfonso Valencia[1,8], María José Rementeria[1], Antonella Santuccione Chadha[3,9], Nikolaos Mavridis[3,10]

[1] *Barcelona Supercomputing Center (BSC), C/ Jordi Girona, 29, 08034, Barcelona, Spain*

[2] *Telefonica Innovation Alpha Health, Torre Telefonica, Plaça d'Ernest Lluch i Martin, 5, 08019, Barcelona, Spain*

[3] *The Women's Brain Project (WBP), Guntershausen, Switzerland*

[4] *Wega Informatik AG, Aeschengraben 20 CH-4051, Basel, Switzerland*

[5] *Research Programme on Biomedical Informatics (GRIB), Hospital del Mar Research Institute and Pompeu Fabra University, Dr. Aiguader, 88, 08003, Barcelona, Spain*

[6] *Eurecat - Centre Tecnològic de Catalunya, C/ Bilbao, 72, Edifici A, 08005, Barcelona, Spain*

[7] *eHealth Center, Universitat Oberta de Catalunya, Rambla del Poblenou, 156, 08018 Barcelona, Spain*

[8] *ICREA, Pg. Lluís Companys 23, 08010, Barcelona, Spain*

[9] *Roche Diagnostics International, Forrenstrasse 2, 6343 Rotkreuz ZG, Switzerland*

[10] *Interactive Robots and Media Laboratory (IRML), United Arab Emirates*

* corresponding author: davide.cirillo@bsc.es (Tel: +34 934137971)
# Contributed equally

Supplementary Notes

1. Sex and gender

Sex is an almost always binary biological variable based on the alternative assignment of an X or Y to the 23rd chromosome [1]. It potentially constitutes the most prominent phenotypic difference distinguishing humans. Clear biological differences between male and females include selective activation of several cellular pathways, affecting aspects such as the longer life span in female, which is attributed to compensation for deleterious mutations on one of the two identical sex chromosomes. Disease prevalence varies substantially across the two sexes, ascribed to differences in the endocrine systems. Moreover, individuals that present abnormalities carrying an atypical number of X and/or Y-chromosomes are defined intersex and display variabilities in their manifestation and vulnerability to diseases.

*Gender***,** on the other hand, entails socially constructed characteristics (norms, roles and relations) of women, men and other non-binary categories [2]. While *gender identity* is a person's inherent sense of self that may or may not correspond to the person's sex, *gender expression* is the presentation of that person, including appearance and behavior that may or may not conform to the person's sex or gender identity [3]. Gender varies across different societies and it can be changed. Individuals and groups that unmeet the established gender standards often face stigma, discrimination or social exclusion. Norms, roles and relations, as well as stigma discrimination and social exclusion affect both mental and physical health and wellbeing. Along with influencing disease susceptibility, these factors affect an individual's access to and uptake of health services and the health outcomes [4].

2. Explainable Artificial Intelligence

When it comes to the explainability, there are different aspects that are expected to be offered by Explainable Artificial Intelligence (XAI) approaches.

Interpretability - The goal is to describe the internals of a system in a way that is understandable to humans. The success of this goal is tied to cognition, knowledge, and biases of the user: for a system to be interpretable, it must produce descriptions that are simple enough for a person to understand using a vocabulary that is meaningful to the user.

Completeness - The goal is to describe the operation of a system in an accurate way. An explanation is more complete when it allows the behavior of the system to be anticipated in more situations.

XAI developments have focused on either explaining the *data processing* or the *data representation*. An explanation of processing is analogous to explaining the execution trace of a program. An explanation about representation can be compared to explaining the internal data structures of a program. A third approach to interpretability is to create *explanation producing systems* with architectures that are designed to simplify interpretation of their own behavior. Such architectures can be designed to make either their processing, representations, or other aspects of their operation easier for people to understand.

Supplementary References

1. Schiebinger, L., Leopold, S. S. & Miller, V. M. Editorial policies for sex and gender analysis. *The Lancet* **388**, 2841–2842 (2016).
2. WHO | Gender. *WHO* http://www.who.int/gender-equity-rights/understanding/gender-definition/en/
3. American Psychological Association. Guidelines for psychological practice with transgender and gender nonconforming people. *Am. Psychol.* **70**, 832–864 (2015).
4. Reisner, S. L. *et al.* Monitoring the health of transgender and other gender minority populations: Validity of natal sex and gender identity survey items in a U.S. national cohort of young adults. *BMC Public Health* **14**, 1224 (2014).
